# Lysophosphatidylcholine Acyltransferase1 Overexpression Promotes Oral Squamous Cell Carcinoma Progression via Enhanced Biosynthesis of Platelet-Activating Factor

**DOI:** 10.1371/journal.pone.0120143

**Published:** 2015-03-24

**Authors:** Tomomi Shida-Sakazume, Yosuke Endo-Sakamoto, Motoharu Unozawa, Chonji Fukumoto, Ken Shimada, Atsushi Kasamatsu, Katsunori Ogawara, Hidetaka Yokoe, Masashi Shiiba, Hideki Tanzawa, Katsuhiro Uzawa

**Affiliations:** 1 Department of Oral Science, Graduate School of Medicine, Chiba University, Chiba, Japan; 2 Department of Dentistry and Oral-Maxillofacial Surgery, Chiba University Hospital, Chiba, Japan; 3 Department of Oral and Maxillofacial Surgery Research Institute, National Defense Medical College, Saitama, Japan; 4 Department of Medical Oncology, Graduate School of Medicine, Chiba University, Chiba, Japan; China Medical University, TAIWAN

## Abstract

**Background:**

The relevance of lysophosphatidylcholine acyltransferase1 (LPCAT1), a cytosolic enzyme in the remodeling pathway of phosphatidylcholine metabolism, in oral squamous cell carcinoma (OSCC) is unknown. We investigated LPCAT1 expression and its functional mechanism in OSCCs.

**Methods:**

We analyzed LPCAT1 mRNA and protein expression levels in OSCC-derived cell lines. Immunohistochemistry was performed to identify correlations between LPCAT1 expression levels and primary OSCCs clinicopathological status. We established LPCAT1 knockdown models of the OSCC-derived cell lines (SAS, Ca9-22) for functional analysis and examined the association between LPCAT1 expression and the platelet-activating factor (PAF) concentration and PAF-receptor (PAFR) expression.

**Results:**

LPCAT1 mRNA and protein were up-regulated significantly (p<0.05) in OSCC-derived cell lines compared with human normal oral keratinocytes. Immunohistochemistry showed significantly (p<0.05) elevated LPCAT1 expression in primary OSCCs compared with normal counterparts and a strong correlation between LPCAT1-positive OSCCs and tumoral size and regional lymph node metastasis. In LPCAT1 knockdown cells, cellular proliferation and invasiveness decreased significantly (p<0.05); cellular migration was inhibited compared with control cells. Down-regulation of LPCAT1 resulted in a decreased intercellular PAF concentration and PAFR expression.

**Conclusion:**

LPCAT1 was overexpressed in OSCCs and correlated with cellular invasiveness and migration. LPCAT1 may contribute to tumoral growth and metastasis in oral cancer.

## Introduction

Lysophosphatidylcholine acyltransferase1 (LPCAT1) is a cytosolic enzyme that catalyzes the conversion of lysophosphatidylcholine (LPC) to phosphatidylcholine (PC) in remodeling the pathway of PC biosynthesis. LPCAT1 is expressed constantly in lung tissue, especially in type II alveolar cells, and plays a fundamental role in generating dipalmitoyl-PC of pulmonary surfactant [1]. To date, LPCAT1 overexpression has been reported in hepatocellular carcinoma [2], colorectal adenocarcinoma [3], and prostate cancer [4–6] and has been described as contributing to cancer progression, metastasis, and recurrence. We previously reported the gene expression profile of OSCC to identify cancer-related genes, and LPCAT was up-regulated in OSCC-derived cell lines [7]. Recently, LPCAT1 was found catalyzing the biosynthesis of platelet-activating factor (PAF) (1-O-alkyl-2-acetyl-sn-glycero-3-phosphorylcholine) with its lyso-PAF acetyltransferase activity [8, 9].

PAF is a lipid mediator involved in numerous biologic responses, including platelet activation, airway construction, and hypotension [9]. In the normal state, PAF is conditioned in very low concentrations, but in cases with some types of stimulation such as inflammation, PAF is produced immediately in several cellular types such as leukocytes, platelets, macrophages, and endothelial and renal cells [10, 11]. PAF is biosynthesized through two independent pathways called the *de novo* and remodeling pathways by lyso-PAF acetyltransferase [8]. There are two lyso-PAF acetyltransferases, LPCAT1 and LPCAT2, both of which produce PAF via remodeling pathway. LPCAT2 is the first detected lyso-PAF acetyltransferase that catalyzes PAF biosynthesis in inflammatory cells such as macrophages, leukocytes, and neutrophils. This enzyme is Ca^2+^ dependent and activated in response to lipopolysaccharide or Toll-like receptor stimulation. In contrast, LPCAT1, recently recognized as another lyso-PAF acetyltransferase, is predominantly expressed in lung tissue and its activity is Ca^2+^ independent. Moreover, LPCAT1 is neither activated nor up-regulated by inflammatory stimulation. Thus, LPCAT1 have been thought to be non-inflammatory/constitutive lyso-PAF acetyltransferase. However, the role of PAF constitutively produced by LPCAT1 was still unknown.

When cells are exposed to PAF, it binds to a specific receptor, PAF receptor (PAFR), which has restricted expression in key target cells of the inflammatory, immune, and hemostatic systems [12, 13]. PAFR belongs to the G protein-coupled receptor protein family, and activated tyrosine kinase transduces cellular signals via Erk [14], Janus kinase 2 [15], RhoA, p38MAPK [16], and other mediators. Activation of the PAF/PAFR pathway induces cellular proliferation in human epithelial cells, skin fibroblasts [17], and pulmonary vascular smooth cell [18]. Recently, numerous studies have evaluated the relation between PAF/PAFR and carcinogenesis and tumoral malignancies and reported some essential effects of PAF in the tumoral microenvironment [19].

In the current study, LPCAT1 was overexpressed in OSCC-derived cell lines and primary OSCCs. We also analyzed the correlation between LPCAT1 expression and clinicopathological characteristics. Furthermore, we assumed that LPCAT1 affects the functional characteristics of OSCC via PAF production and performed functional analysis to define the biologic effects and molecular mechanism of LPCAT1.

## Materials and Methods

### Ethical statement

The Ethical Committee of the Graduate School of Medicine, Chiba University approved the study protocol (approval number, 236), which was performed in accordance with the ethical standards of the Declaration of Helsinki. All patients provided written informed consent.

### OSCC-derived cell lines and tissue samples

RIKEN (Ibaraki, Japan) provided the Sa3, HO-1-u-1, KOSC-2, Ca9–22, HO-1-N-1, HSC-2, and HSC-3 cell lines through the National Bio-Resource Project of the Ministry of Education, Culture, Sports, Science and Technology, Tokyo, Japan. Short tandem repeat profiles confirmed the cellular identity. All OSCC-derived cells were grown in Dulbecco’s modified Eagle’s medium (DMEM) (Sigma, St. Louis, MO, USA) supplemented with 10% fetal bovine serum (FBS) (Sigma) and 50 units/ml of penicillin and streptomycin (Sigma). Primary cultured human normal oral keratinocytes (HNOKs) were used as normal controls [20, 21]. The HNOKs were healthy oral mucosal epithelial specimens collected from young patients aged 22–34 at Chiba University Hospital. These independent HNOKs were primary cultured and maintained in Keratinocyte-SFM (Gibco, Life Technologies, Carlsbad, CA, USA) comprised of 5 ml of oral keratinocyte growth supplement (ScienCell Research Laboratories, Carlsbad, CA, USA) and 5ml of penicillin/streptomycin solution (ScienCell Research Laboratories).

Fifty-five pairs of primary OSCCs samples and corresponding normal oral epithelial tissues were obtained intraoperatively at Chiba University Hospital. The resected tissues were divided for RNA isolation and immunohistochemistry (IHC); the former tissues were frozen immediately and stored at -80°C, the latter tissues were fixed in 20% buffered formaldehyde solution. Each tissue specimen was diagnosed histopathologically according to the World Health Organization criteria by the tumor-node-metastases classification of the International Union against Cancer. All OSCC samples were confirmed histologically that tumor was present in over 90% of the specimens.

### Preparation of cDNA and protein

Total RNA was isolated using TRIzol Reagent (Invitrogen, Carlsbad, CA, USA), according to the manufacturer’s instructions. cDNA was generated from 1 μg of total RNA using ReverTra Ace (TOYOBO CO., LTD., Osaka, Japan), according to the instruction manual. The cells were washed twice with cold phosphate-buffered saline (PBS) and centrifuged briefly. The cell pellets were incubated at 4°C for 30 min in a lysis buffer (7 M urea, 2 M thiourea, 4% [w/v] CHAPS, and 10 mM Tris, pH 7.4) with a proteinase inhibitor cocktail (Roche Diagnostics, Mannheim, Germany). The protein concentration was measured using a commercial Bradford reagent (BioRad, Richmond, CA, USA).

### mRNA expression analysis

Real-time quantitative reverse transcriptase-polymerase chain reaction (qRT-PCR) was performed to evaluate the expression level of the target gene (*LPCAT1*) in the OSCC-derived cell lines and HNOKs. qRT-PCR was performed with one method using LightCycler 480 Probes Master kit (Roche Diagnostics). Primers were designated using the ProbeFinder qPCR assay design software accessible at the Universal ProbeLibrary (http://qpcr.probefinder.com/roche3.html). The nucleotide sequences of gene-specific primers for qRT-PCR amplification were used: *LPCAT1*, forward, 5’-CACAACCAAGTGGAAATCGAG-3’; and reverse 5’-GCACGTTGCTGGCATACA-3’, (universal probe #35). All qRT-PCR analyses were performed using the LightCycler 480 PCR system (Roche Diagnostics). The reaction mixture was loaded onto a PCR plate and subjected to an initial denaturation at 95°C for 10 minutes, followed by 60 cycles of amplification, at 95°C for 10 seconds for denaturation, at 60°C for 30 seconds for primer annealing, and 72°C for 1 second for extension, followed by a cooling step at 50°C for 30 seconds. The transcript amounts for the target genes were estimated from the respective standard curves and normalized to the glyceraldehyde-3-phospate dehydrogenase (*GAPDH*, forward, 5’-CATCTCTGCCCCCTCTGCTGA-3’; reverse, 5’-GGATGACCTTGCCCACAGCCT-3’; and universal probe #60). The transcript amount for LPCAT1 was estimated from the respective standard curves and normalized with the GAPDH transcript amount determined in corresponding samples.

### Immunoblot analysis

Protein extracts (20 μg) were electrophoresed in 4–12% Bis-Tris gel (Invitrogen), transferred to polyvinylidene difluoride membranes (Invitrogen), and blocked for 1 hour at room temperature in Blocking One (Nacalai Tesque Kyoto, Japan). The membrane were washed three times with 0.1% Tween-20 in Tris-buffered saline and incubated with 1.0 μg/ml affinity-purified rabbit anti-human LPCAT1 polyclonal antibody (Proteintech, Chicago, IL, USA) (1:1000 dilution in TBS-T), and goat anti-PAFR polyclonal antibody (Santa Cruz Biotechnology. Inc., CA, USA) (1:100 dilution in TBS-T) overnight at 4°C. The membranes were washed with 0.1% Tween20 in Tris-buffered saline and incubated with a secondary antibody and coupled to horseradish peroxidase-conjugated anti-rabbit or anti-goat IgG (Promega, Madison, WI, USA) for 1 hour at room temperature. Finally, the membranes were detected using SuperSignal West Pico Chemiluminescent substrate (Thermo Fischer Scientific, Rockford, IL, USA) and immunoblotting was visualized by exposing the membranes to ATTO Light-Capture II (ATTO, Tokyo, Japan). Signal intensities were quantitated using the CS Analyzer version 3.0 software (ATTO). Densitometric LPCAT1 protein data were normalized to GAPDH protein levels.

### IHC

IHC was performed on 4-μm sections of paraffin-embedded specimens using rabbit anti-LPCAT1 polyclonal antibody (Proteintech) or anti-LPCAT2 polyclonal antibody (Santa Cruz Biotechnology). Briefly, after deparaffinization and hydration, the endogenous peroxidase activity was quenched by a 30-minute incubation in a mixture of 3% hydrogen peroxide solution (diluted in distilled water), after which the sections were blocked for 1 hour at room temperature with 1.0% bovine serum albumin in PBS before reaction overnight with anti LPCAT1 antibody at 4°C in a moist chamber. Upon incubation with the primary antibody, the specimens were washed three times with PBS and treated with Envision reagent (DAKO, Carpentaria, CA, USA) or HRP-rabelled rabbit anti-goat IgG polycronal antibody (Abcam Ltd. Cambridge UK) followed by color development in 3,3’-diaminobenzine tetrahydrochloride (DAKO). The slides then were counterstained lightly with hematoxylin, dehydrated with ethanol, cleaned with xylene, and mounted with Malinol (Muto Pure Chemicals Co., Tokyo, Japan). In order to confirm the reaction of antibody, we stained mouse lung tissues and mouse pancreatic tissue as positive controls for LPCAT1 and LPCAT2 respectively. While, non-specific bindings of an antibody to proteins other than the antigen sometimes occurred. As a negative control, the slides were immunostained without exposure to primary antibodies, which confirmed the staining specificity. To quantify the status of the LPCAT1 protein expression in those components, we used an IHC scoring system described previously [22–26]. This scoring system was established for semiquantitative evaluation of IHC staining. The intensities of the LPCAT1 immunoreaction in the cell were scored as follows: 1+, weak; 2+, moderate; and 3+, intense. The cellular numbers and the staining intensities then were multiplied to produce a LPCAT1 IHC score. Cases with a LPCAT1 IHC score exceeding 61.8 (+3 standard deviation [SD] score for normal tissue) were defined as LPCAT1-positive. The ±3-SD cutoff, which statistically is just 0.2% of the measurement and is expected to fall outside this range, was used because it was unlikely to be affected by a random experimental error produced by a sample manipulation [27]. Two independent pathologists, neither of whom had knowledge of the patients’ clinical status, made these judgments.

### Transfection with shRNA plasmid

OSCC-derived cells (SAS and Ca9–22) were stably transfected with the LPCAT1 shRNA (shLPCAT1, Santa Cruz Biotechnology) or the control shRNA (shMock, Santa Cruz Biotechnology) construct by Lipofectamine LTX and Plus Reagents (Invitrogen). After transfection, the stable transfectants were isolated by a culture medium containing 2μg/ml of Puromycin (Invitrogen). Two to three weeks after transfection, viable colonies were transferred to new dishes. shLPCAT1- and shMock-transfected cells were used for further experiments.

### Proliferation assay

To evaluate the effect of LPCAT1 knockdown on cellular proliferation, we analyzed cellular growth in shLPCAT1- and shMock-transfected cells. These transfectants were seeded in 6-cm dishes at a density of 1×10^4^ viable cells per dishes. The experiments were carried out for 192 hours by counting the cells every 24 hours. At the indicated time points, the cells were trypsinized and counted using a hemocytometer in triplicate samples. We compared the numbers of the shLPCAT1- and shMock-transfected cells.

### Invasiveness assay

To evaluate the effect of LPCAT1 knockdown on invasiveness, a total of 2.5×10^5^ cells resuspended in the serum-free medium were seeded on a 0.8-μm polyethylene terephthalate membrane insert in a transwell apparatus (Becton-Dickinson Labware, Franklin Lakes, NJ, USA). In the lower chamber, 2 ml of DMEM with 10% FBS was added as a chemoattractant. After the cells were incubated for 48 hours at 37°C, the insert was washed with PBS, and the cells on the top surface of the insert were removed with cotton swabs. Cells adhering to the lower surface of the membrane were fixed with methanol and stained with crystal violet. The number of cells that invaded through the pores in five random fields was counted using a light microscope at ×100 magnification.

### Wound healing assay

To evaluate the effect of LPCAT1 knockdown on migration, we performed a wound healing assay as described previously [28, 29]. Briefly, after uniform wounds were made in confluent culture of shLPCAT1- and shMock-transfected cells, the extent of closure was monitored visually every 3 hours for 24 hours. The results were visualized by measuring the wound spaces using the Lenaraf220 software (http://www.vector.co.jp/soft/dl/win95/art/se312811.html). The mean value was calculated from data from three separate chambers.

### ELISA of PAF

To assess the effect of LPCAT1 knockdown on PAF production, we performed an enzyme-linked immunosorbent assay (ELISA) on PAF using an ELISA kit for PAF (Cloud-Clone Corp, Houston, TX, USA), according to the instruction manual with some modification. Briefly, after cells were cultured to confluence in serum-supplemented DMEM and washed three times with ice-cold PBS and then scraped off. The harvested cells were diluted with 500 μl of PBS and the cellular membranes were broken by a freezing and thawing method. The cellular lysates were centrifuged for 5 minutes at 5,000 g in 4°C, and the collected supernatants were assayed. This ELISA kit used the competitive inhibition enzyme technique, and absorbance at 450 nm was determined with microplate reader (Benchmark Plus, Bio-Rad, Hercules, CA, USA). To standardize the values, we used a protein concentration of samples as an internal control.

### Statistical analysis

All statistical analyses were performed using the Microsoft Excel (Microsoft, Redmond, WA, USA). The statistical significance of the LPCAT1 expression levels was evaluated using the Mann-Whitney U-test. Fischer’s exact tests were used to compare categorical variables. All tests were two-sided. P<0.05 was considered statistically significant. The data are expressed as the mean ±standard error of the mean.

## Results

### LPCAT1 mRNA and protein up-regulation in OSCC-derived cell lines

**Fig 1:**
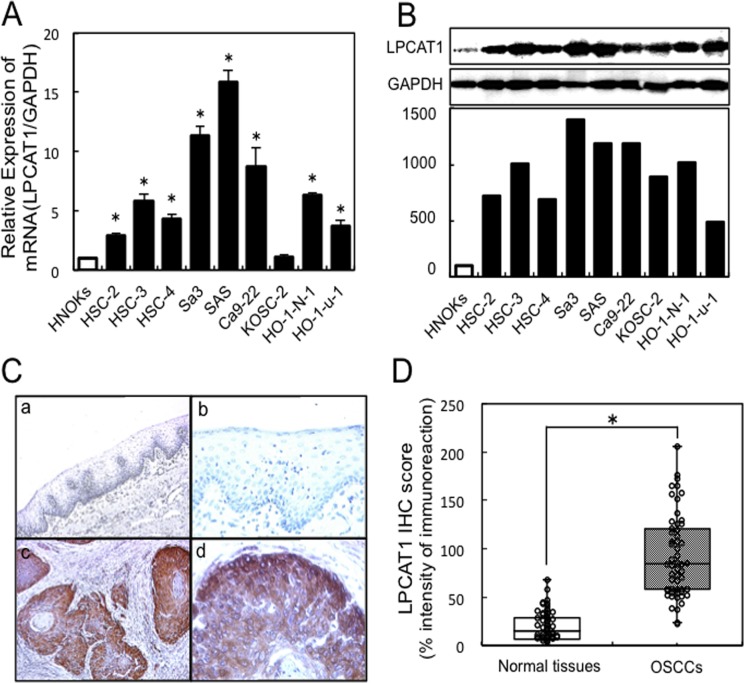
Expression profiles of LPCAT1 in OSCC-derived cell lines and OSCC samples. (**A**) Quantification of *LPCAT1* mRNA levels in OSCC-derived cell lines by qRT-PCR analysis. To determine the mRNA expression status of *LPCAT1*, we performed qRT-PCR analysis using 9 OSCC-derived cell lines (HSC-2, HSC-3, HSC-4, Sa3, SAS, Ca9–22, KOSC2, HO-1-N-1, and HO-1-u-1), and HNOKs. *LPCAT1* mRNA is significantly up-regulated in the nine OSCC-derived cell lines compared with the HNOKs. The data are expressed as the mean ±SEM of values from three assays (*p<0.05, Mann-Whitney *U* test). (**B**) Immunoblot analysis of LPCAT1 in the OSCC-derived cells lines and HNOKs. To investigate the protein expression status of LPCAT1, we performed immunoblot analysis in the same OSCC-derived cell lines and HNOKs. The LPCAT1 protein expression level is significantly up-regulated in all OSCC-derived cell lines compared with the HNOKs. Densitometric LPCAT1 protein data are normalized to the GAPDH protein levels. The values are expressed as a percentage of the HNOKs. (**C**) IHC of LPCAT1 on primary OSCC samples. Representative IHC results are shown for LPCAT1 protein in normal oral tissue (a, b) and primary OSCCs (c, d). The original magnifications are 100×(a, c), and 400×(b, d). Strong LPCAT1 immunoreactivity is detected in the primary OSCCs. (**D**) The status of LPCAT1 protein expression in primary OSCCs (n = 55) and the normal counterparts. The LPCAT1 IHC scores are calculated as follows: IHC score = 1×(number of weakly stained cells in the field) + 2×(number of moderately stained cells in the field) + 3×(number of intensely stained cells in the fields). The LPCAT1 IHC scores for normal oral tissues range from 0.5 to 68.5 and that of primary OSCCs range from 23.7 to 205.9. The LPCAT1 protein expression levels in OSCCs are significantly (*p<0.01, Mann-Whitney *U* test) higher than those in normal oral tissues.

To analyze the LPCAT1 expression status, we performed qRT-PCR and immunoblotting analysis using OSCC-derived cell lines (HSC-2, HSC-3, HSC-4, Ca9–22, KOSC2, SAS, Sa3, HO-1-u1, and HO-1-N1) and HNOKs. *LPCAT1* mRNA was significantly (p<0.05) up-regulated in almost all OSCC-derived cell lines except for KOSC2 compared with the HNOKs (Fig. 1A). Fig. 1B shows representative results of immunoblotting analysis of LPCAT1 protein expression compared with the HNOKs. A significant increase in LPCAT1 protein expression was seen in all OSCC-derived cell lines compared with the HNOKs. Expression analysis indicated that translational products of this molecule were highly expressed in OSCC-derived cell lines.

#### Overexpression of LPCAT1 in primary OSCCs

To determine the LPCAT1 expression status in primary OSCCs and its relevance to the clinicopathological characteristics, we analyzed the LPCAT1 protein expression in primary OSCCs and paired normal oral tissues from 55 patients using the IHC scoring system. We also examined IHC for LPCAT2 protein expression in primary OSCCs, but there was no significant immunoreaction (S1 Fig.). Fig. 1C shows representative IHC results for LPCAT1 protein in normal oral tissues and primary OSCCs. Strong LPCAT1 immunoreactivity was detected in the cytoplasm in the OSCCs (Fig. 1C: c, d), while the normal tissues showed negative immunostaining (Fig. 1C: a, b). The LPCAT1 IHC scores ranged from 23.7 to 205.9 in OSCCs (median, 84.5) and from 0.5 to 68.5 in normal counterparts (median, 15.17). The IHC scores in primary OSCCs were significantly (p<0.05) higher than those in the normal oral tissues (Fig. 1D). After statistical analysis, 36 (65%) of 55 OSCC samples were considered LPCAT1-positive. We then analyzed the correlations between the clinicopathological characteristics of the patients with OSCC and the status of the LPCAT1 protein expression using the IHC scoring system (Table 1). Among the clinical classifications, the LPCAT1-positive OSCCs were correlated significantly (p<0.05 for all comparisons) with larger tumors, frequent regional lymph node metastasis, and advanced clinical stages. No significant relations were found with age, gender, histopathological type, or tumoral site.

**Table 1:** Correlation between clinicopathological parameters of patients with OSCC and LPCAT1 protein expression.

	Result of immunostaining					
Parameter	No. of patients/ (%)					
	Total	LPCAT1(-)		LPCAT1(+)		*p* value
Age at surgery (year)						
<60	14	4	(29%)	10	(71%)	0.816
60~70	16	5	(31%)	11	(69%)	
>70	25	10	(40%)	15	(60%)	
Gender						
Male	34	11	(32%)	23	(68%)	0.663
Female	21	8	(38%)	13	(62%)	
T-primary tumor						
1	2	1	(50%)	1	(50%)	0.005 *
2	34	17	(50%)	17	(50%)	
3	11	1	(9%)	10	(91%)	
4	8	0	(0%)	8	(100%)	
N-regional lymph node						
–	32	16	(50%)	16	(50%)	0.009 *
+	23	3	(13%)	20	(87%)	
Stage						
I	2	1	(50%)	1	(50%)	0.001 *
II	24	14	(58%)	10	(42%)	
III	11	3	(27%)	8	(73%)	
IV	18	1	(6%)	17	(94%)	
Histopathologic type						
Well	37	12	(32%)	25	(68%)	0.811
Moderate	14	5	(36%)	9	(64%)	
Poor	4	2	(50%)	2	(50%)	
Tumor site						
Tongue	35	14	(40%)	21	(60%)	0.263
Gingiva	15	2	(13%)	13	(87%)	
Buccal mucosa	2	0	(0%)	2	(100%)	
Soft palate	2	2	(100%)	0	(0%)	
Oral floor	1	1	(100%)	0	(0%)	

* *p*<0.05

### Establishment of LPCAT1 knockdown cells

**Fig 2:**
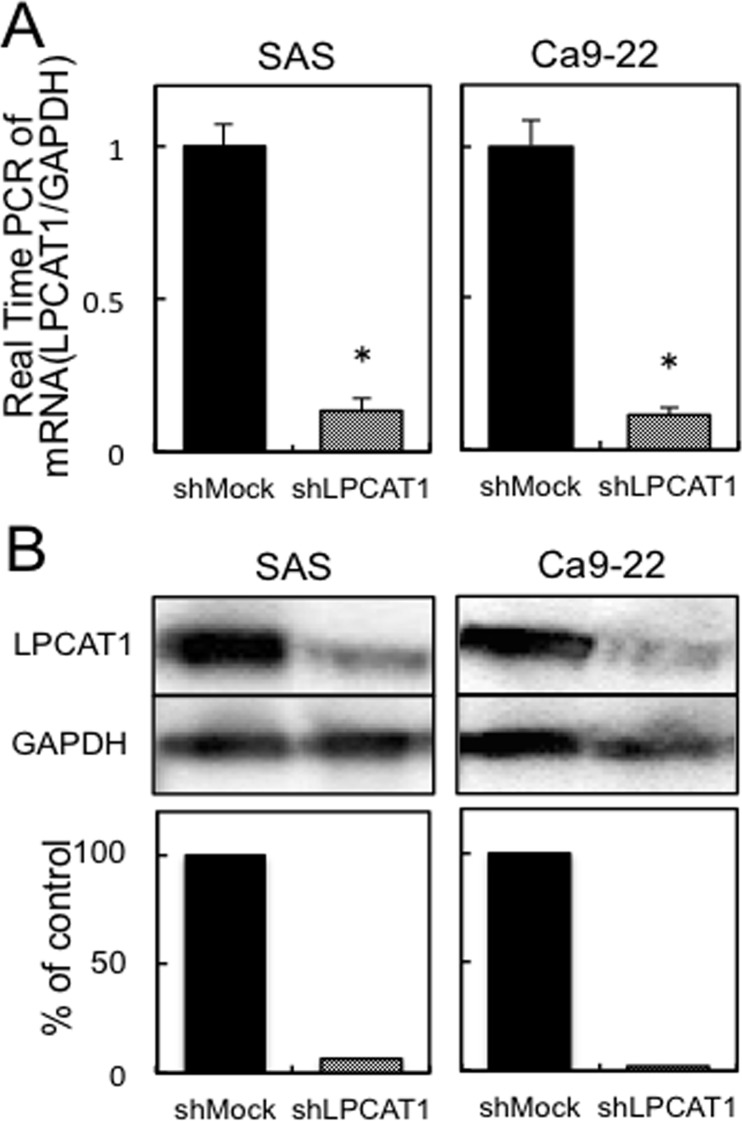
Establishment of shLPCAT1-transfected cells. (**A**) Expression of *LPCAT1* mRNA in shMock- and shLPCAT1-transfected cells (SAS- and Ca9–22-derived transfectants). *LPCAT1* mRNA expression in shLPCAT1-transfected cells is significantly (*p<0.05, Mann-Whitney *U* test) lower than in the shMock-transfected cells. (**B**) Immunoblot analysis of LPCAT1 protein in shMock- and shLPCAT1-transfected cells (SAS- and Ca9–22-derived transfectants). The LPCAT1 protein expression in shLPCAT1-transfected cells is decreased markedly compared with the shMock-transfected cells.

To investigate the LPCAT1 function in vitro, we established LPCAT1 knockdown cells using shRNA system. LPCAT1 shRNA (shLPCAT1) and the control shRNA (shMock) were transfected in the OSCC-derived cell lines, SAS and Ca9–22, respectively. The expression levels of LPCAT1 mRNA and protein in shLPCAT1-transfected cells were significantly (p<0.05) lower than those in shMock-transfected cells (Fig. 2A, B).

### Decreased cellular proliferation, migration, invasiveness in LPCAT1 knockdown cells

**Fig 3:**
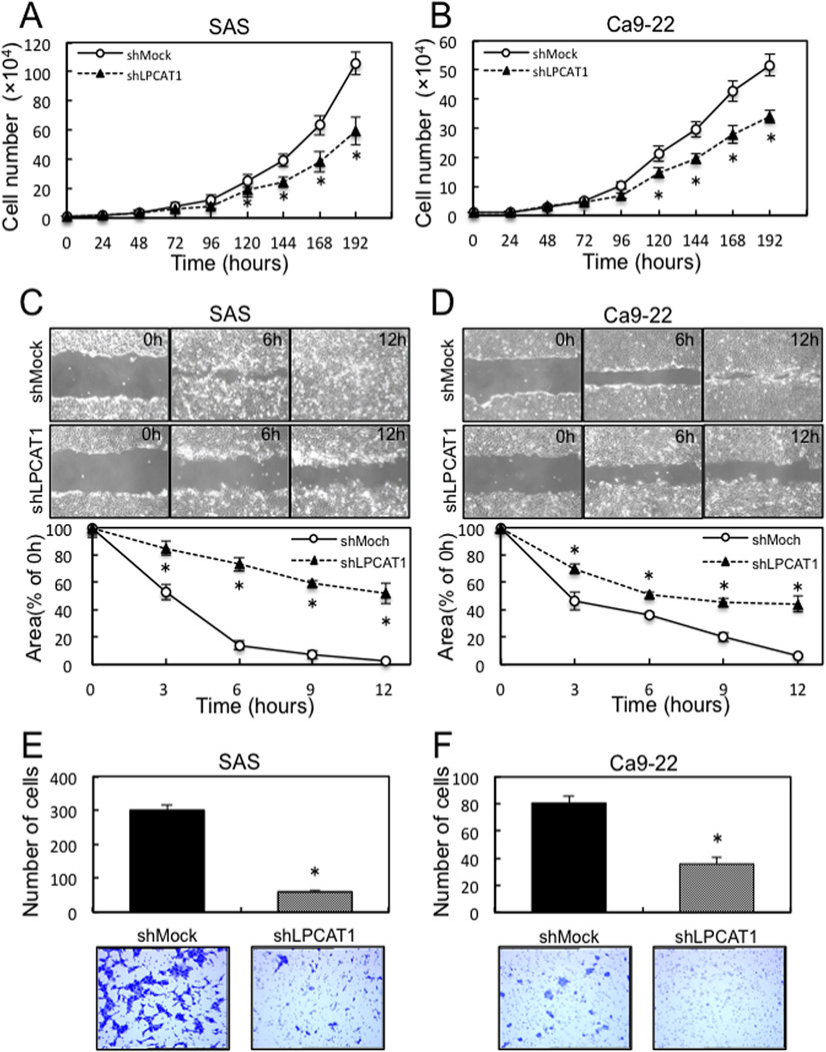
Effect of LPCAT1 knockdown on OSCC-derived cell lines. (**A, B**) Proliferation assay of shMock- and shLPCAT1-transfected cells (SAS- and Ca9–22-derived transfectants). To determine the effect of shLPCAT1 on cellular proliferation, shLPCAT1- and shMock-transfected cells were seeded in 6-cm dishes at a density of 1×10^4^ viable cells/well. Both transfected cells were counted on seven consecutive days. The cellular growth of shLPCAT1-transfected cells (SAS- and Ca9–22- derived transfectants) is inhibited significantly compared with the shMock-transfected cells after 5 days (120 hours). The results are expressed as the mean ±SEM of values from three assays. The asterisks indicate significant (*p<0.05, Mann-Whitney *U* test) differences between the shLPCAT1- and shMock-transfected cells. (**C, D**) Migration assay of shMock- and shLPCAT1-transfected cells (SAS- and Ca9–22-derived transfectants). To evaluate the effect of LPCAT1 knockdown on migration, uniform wounds were made in confluent culture of the shLPCAT1- and shMock-transfected cells (SAS- and Ca9–22-derived transfectants) and the extent of closure was monitored visually every 3 hours for 24 hours. The mean value was calculated from data obtained from three separate chambers. The wound area was decreased significantly (*p<0.05, Mann-Whitney *U* test) in the culture of shMock-transfected cells after 12 hours, whereas a gap remained in the shLPCAT1-transfected cells. (**E, F**) Invasiveness assay of shMock- and shLPCAT1-transfected cells (SAS- and Ca9–22-derived transfectants). To evaluate the effect of LPCAT1 knockdown on invasiveness, we seeded 2.5×10^5^ cells in the serum-free medium of a 0.8-μm polyethylene terephthalate membrane insert in a transwell apparatus and added serum-supplemented medium in the lower chamber as a chemoattractant. After incubation at 37°C for 48 hours, cells that penetrated through the pores were fixed, stained, and counted using a light microscope at ×100 magnification. The mean value was calculated from data obtained from three separate chambers. The number of shLPCAT1-transfected cells penetrating through the pores is decreased significantly (*p<0.05, Mann-Whitney *U* test) compared with the shMock-transfected cells.

To investigate the effect of LPCAT1 on cellular proliferation, we monitored cellular growth for 168 hours. SAS and Ca9–22 shLPCAT1-transfected cells had significant (p<0.05) decreases in cellular growth compared with the shMock-transfected cells (Fig. 3A, B). We also performed cellular migration and invasiveness assays to study the biologic effects of LPCAT1 in relation to metastatic capability. In a migration assay, when we visually monitored the area of uniform wounds in confluent cell culture, the wounds in the shLPCAT1-transfected cells closed later than those in the shMock-transfected cells in both cell lines (Fig. 3C, D). In the invasiveness assay, the number of penetrating shLPCAT1-transfected cells decreased compared with shMock-transfected cells (Fig. 3E, F). Therefore, shLPCAT1-transfected cells showed decreased migration and invasiveness capabilities.

### Knockdown of LPCAT1 and suppressed PAF synthesis and PAFR expression in OSCC-derived cell lines

**Fig 4:**
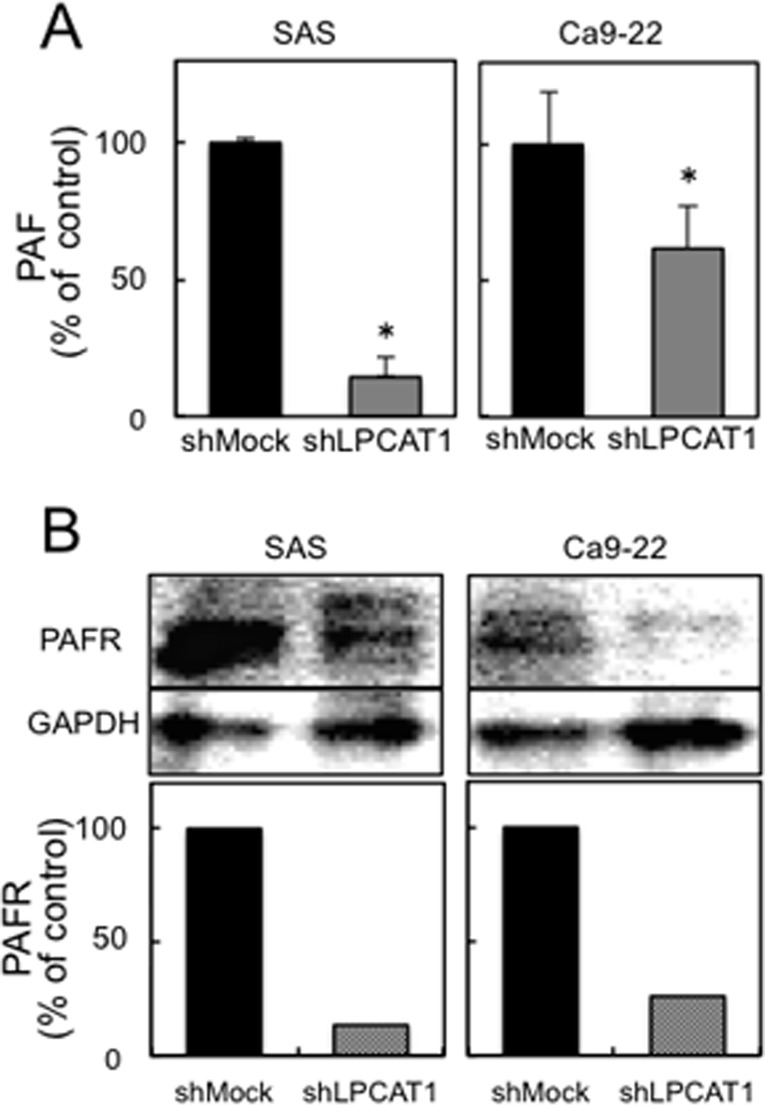
Reduced PAF synthesis and PAFR expression in shLPCAT1-transfected cells. (**A**) Evaluation of intracellular PAF concentration in shMock- and shLPCAT1-transfected cells (SAS- and Ca9–22-derived transfectants). To determine the intercellular PAF concentrations, we examined cellular lysates using ELISA and normalized with protein concentration. The mean value was calculated from the data obtained from three independent samples. The relative PAF concentration in the shLPCAT1-transfected cells is decreased significantly (*p<0.05, the Mann-Whitney *U* test) compared with the shMock-transfected cells. (**B**) Evaluation of PAFR expression in shMock- and shLPCAT1-transfected cells (SAS- and Ca9–22-derived transfectants). Immunoblot analysis shows that PAFR protein expression in the shLPCAT1-transfected cells is decreased markedly compared with the shMock-transfected cells.

The biosynthesis of PAF has been studied extensively in various cells and tissues, and LPCAT1 catalyzed PAF biosynthesis [8]. Numerous studies also have been performed to investigate the effects of PAF on tumoral characteristics [19]. To explain the relation between overexpressed LPCAT1 and cancer malignancy, we investigated the intracellular PAF concentration. To determine the PAF concentrations in shLPCAT1- and shMock-transfected cells, ELISA was carried out using cell lysates. The results showed that the intracellular PAF concentration decreased significantly (p<0.05) in shLPCAT1-transfected cells compared with shMock-transfected cells (Fig. 4A). The levels of intracellular PAF were represented as the normalized index, which was standardized by protein concentration and calculated as the percentage of the PAF concentration relative to that in the shMock-transfected cells.

PAF affects cellular function via binding to and activating PAFR, and PAFR is also overexpressed in response to PAF stimulation [19]. Considering these findings, we conducted further immunoblotting analysis on shLPCAT1- and shMock-transfected cells to detect the status of the PAFR expression. The results showed that LPCAT1 knockdown caused markedly decreased levels of PAFR protein expression (Fig. 4B). Thus, the intracellular PAF concentration and PAFR expression were down-regulated in shLPCAT1-transfected cells.

## Discussion

In the current study, we report here that LPCAT1 often is overexpressed in OSCC-derived cell lines and primary OSCC specimens. Moreover, LPCAT1 protein expression levels in primary OSCCs were correlated significantly (p<0.05) with tumoral size, regional lymph node metastasis, and clinical stages. Functional analysis using LPCAT1 knockdown cells showed that down-regulation of LPCAT1 repressed not only cellular proliferation but also invasiveness and migration. These findings supported the prospect that overexpressed LPCAT1 may contribute to tumoral growth and metastasis in OSCCs. Past studies have reported overexpressed LPCAT1 in cancers and mentioned the close interaction between overexpressed LPCAT1 and malignancies, such as vigorous tumoral growth, frequent metastasis, early recurrence, and poor prognosis [2–6]. However, the molecular mechanisms and detailed function of LPCAT1 in OSCC progression remained unclear.

Xu *et al*. (2013) reported a relationship between LPCAT1 and PAF in prostatic cancers and suggested that PAF may play an important role in accelerating progression of aggressive phenotypes. PAF is a phospholipid mediator with pleotropic and potent biologic effects and functions through binding to and activating its specific receptor PAFR[30, 31]. Until now, PAF and PAFR were extensively studied in relation to carcinogenesis and malignancies [32–36]. PAFR-dependent pathways are activated during experimental tumoral growth, modifying the microenvironment and the phenotype of the tumoral macrophages in ways that favor tumoral growth [32]. The activated endothelium and/or cancer cells are thought to introduce PAF into the tumoral microenvironment [19, 33]. PAFR induces activation of G-proteins and tyrosine kinases, and the signals are transduced to downstream pathways, including NFκB, MAPKs, AKT, PI3, and Src [19, 35]. NFκB enhances tumoral metastasis and augments angiogenesis through activation of matrix metalloproteases (MMPs) and vascular endothelial growth factor [37–41]. PAF also directly activates endothelial cells, causes angiogenesis, and promotes vascular permeability leading to metastasis [35]. PAF/PAFR decreased PTEN activity, leading to phosphorylation of AKT and MAPKs. AKT plays a central role in various oncogenic processes including cellular growth, proliferation, motility, and epithelial mesenchymal transition (EMT) [38, 42]. PAF-induced activation of MAPKs, p38 and ERK1/2, occurred via MMP-dependent cleavage of heparin-binding epidermal growth factor and subsequent activation of the EGF receptor, increase proliferation [35, 43]. Thus, the PAF/PAFR pathway causes cellular growth, proliferation, motility, EMT, and angiogenesis in cancers. Furthermore, PAF can activate cancer cells and endothelial cells to amplify PAF production and PAFR expression on their membranes, in autocrine, endocrine, paracrine and juxtracrine interactions [19].

We thus assumed that the interaction between LPCAT1 with activated PAF/PAFR affects the cellular characteristics in OSCC-derived cells and examined the intracellular PAF concentration using ELISA and PAFR expression using immunoblot analysis. Consistent with our hypothesis, the PAF concentration decreased significantly in LPCAT1 knockdown cells compared with control cells, which suggested that intracellular PAF synthesis may be restricted due to down-regulation of LPCAT1. Moreover, immunoblot analysis showed that PAFR expression also was down-regulated markedly in LPCAT1 knockdown cells. These findings indicated that PAFR amplification weakened because of reduced PAF synthesis and stimulation in LPCAT1 knockdown cells; previous studies reported a similar tendency [6, 19]. Therefore, an intervention in PAF/PAFR pathway activation was possible in relation to overexpression of LPCAT1 and promotion of tumoral growth, invasiveness, and migration in OSCC-derived cell lines.

In conclusion, our data indicated that LPCAT1 might be associated with tumoral progression and metastasis by synthesis of PAF and follow up-regulation of PAFR expression in OSCC. Although further studies are needed to understand the interaction between LPCAT1 and the PAF/PAFR pathway and its functions in the cancer microenvironment, LPCAT1 is a potential biomarker of aggressive tumoral progression in human primary OSCCs.

## Supporting Information

S1 Fig Expression profiles of LPCAT2 in OSCC samples. (**A**) IHC of LPCAT2 on primary OSCC samples. Representative IHC results are shown for LPCAT2 protein in positive control (mouse pancreatic tissue) (a), normal oral tissue (b) and primary OSCCs (c). The original magnifications are 400×(a), 100×(b, c). There are only weak immunoreactions in both of normal oral tissues and primary OSCCs in comparison with positive control. (**B**) The status of LPCAT2 protein expression in primary OSCCs (n = 30) and the normal counterparts (n = 30). IHC scores of LPCAT2 were calculated and its states are shown in the chart. The LPCAT2 IHC scores for normal oral tissues range from 1.83 to 22.33 and that of primary OSCCs range from 2.67 to 23.00. There is no significant difference between LPCAT2 protein expression levels in OSCCs and those in normal oral tissues (*p* = 0.191).
(TIFF)
