# Supplementary figures and images for: Lysophosphatidylcholine Acyltransferase1 Overexpression Promotes Oral Squamous Cell Carcinoma Progression via Enhanced Biosynthesis of Platelet-Activating Factor
Source: PLoS One. 2015 Mar 24;10(3):e0120143. doi: 10.1371/journal.pone.0120143 (PMC4372572; doi:10.1371/journal.pone.0120143)

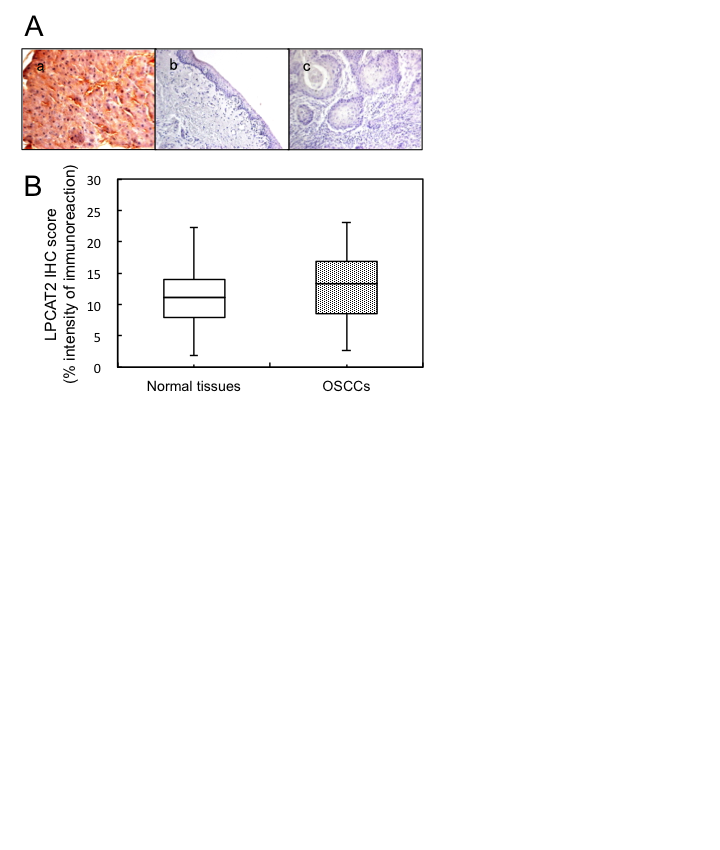

Supplement: S1 Fig — (A) IHC of LPCAT2 on primary OSCC samples. Representative IHC results are shown for LPCAT2 protein in positive control (mouse pancreatic tissue) (a), normal oral tissue (b) and primary OSCCs (c). The original magnifications are 400×(a), 100×(b, c). There are only weak immunoreactions in both of normal oral tissues and primary OSCCs in comparison with positive control. (B) The status of LPCAT2 protein expression in primary OSCCs (n = 30) and the normal counterparts (n = 30). IHC scores of LPCAT2 were calculated and its states are shown in the chart. The LPCAT2 IHC scores for normal oral tissues range from 1.83 to 22.33 and that of primary OSCCs range from 2.67 to 23.00. There is no significant difference between LPCAT2 protein expression levels in OSCCs and those in normal oral tissues (p = 0.191). (TIFF) [file pone.0120143.s001.tiff]
